# Chigger mite (*Eutrombicula alfreddugesi*) ectoparasitism does not contribute to sex differences in growth rate in eastern fence lizards (*Sceloporus undulatus*)

**DOI:** 10.1002/ece3.10590

**Published:** 2023-10-11

**Authors:** Hailey Conrad, Nicholas B. Pollock, Henry John‐Alder

**Affiliations:** ^1^ Department of Ecology, Evolution, and Natural Resources Rutgers University New Brunswick New Jersey USA; ^2^ Graduate Program in Ecology and Evolution Rutgers University New Brunswick New Jersey USA; ^3^ Rutgers Pinelands Field Station Rutgers University New Lisbon New Jersey USA; ^4^ Present address: Department of Biological Sciences Virginia Polytechnic Institute and State University Blacksburg Virginia USA; ^5^ Present address: Department of Biology University of Texas at Arlington Arlington Texas USA

**Keywords:** body size, mite load, parasites, sexual size dimorphism, SSD, tolerance

## Abstract

Parasitism is nearly ubiquitous in animals and is frequently associated with fitness costs in host organisms, including reduced growth, foraging, and reproduction. In many species, males tend to be more heavily parasitized than females and thus may bear greater costs of parasitism. *Sceloporus undulatus* is a female‐larger, sexually size dimorphic lizard species that is heavily parasitized by chigger mites (*Eutrombicula alfreddugesi*). In particular, the intensity of mite parasitism is higher in male than in female juveniles during the period of time when sex differences in growth rate lead to the development of sexual size dimorphism (SSD). Sex‐biased differences in fitness costs of parasitism have been documented in other species. We investigated whether there are growth costs of mite ectoparasitism, at a time coinciding with sex differences in growth rate and the onset of SSD. If there are sex‐biased growth costs of parasitism, then this could suggest a contribution to the development of SSD in *S. undulatus*. We measured growth and mite loads in two cohorts of unmanipulated, field‐active yearlings by conducting descriptive mark‐recapture studies during the activity seasons of 2016 and 2019. Yearling males had consistently higher mid‐summer mite loads and consistently lower growth rates than females. However, we found that growth rate and body condition were independent of mite load in both sexes. Furthermore, growth rates *and* mite loads were higher in 2019 than in 2016. Our findings suggest that juveniles of *S. undulatus* are highly tolerant of chigger mites and that any costs imposed by mites may be at the expense of functions other than growth. We conclude that sex‐biased mite ectoparasitism does not contribute to sex differences in growth rate and, therefore, does not contribute to the development of SSD.

## Graphical abstract

We tested whether a sex‐biased growth cost of parasitism contributes to the development of SSD in Sceloporus undulatus, a female‐larger lizard species. Yearling males had higher mite loads and lower growth rates than females, but growth rate and body condition were independent of mite load in both sexes. We conclude that sex‐biased mite ectoparasitism does not contribute to sex differences in growth rate and the development of SSD.

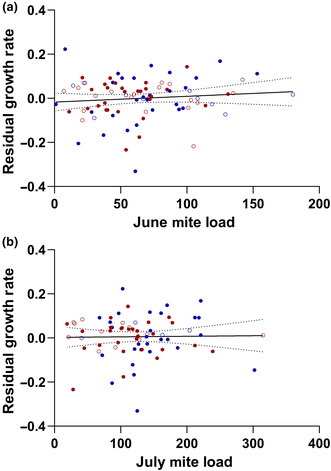

## 1 INTRODUCTION

Parasitism is nearly ubiquitous in animals (Dobson et al., 2008) and is frequently associated with fitness costs in the host organism (Albery et al., 2021; Hicks, Burthe, Daunt, Newell, Butler, et al., 2018; Wittman & Cox, 2021). Sub‐lethal costs of parasitism can reduce host fitness by reducing energy expenditure on growth, foraging, or reproduction (Booth et al., 1993; Gooderham & Shulte‐Hostedde, 2011; Hicks, Burthe, Daunt, Newell, Butler, et al., 2018; Hicks, Burthe, Daunt, Newell, Chastel, et al., 2018; Lehmann, 1993; Lin et al., 2014; Martin et al., 2003; Reed et al., 2008). Furthermore, broad‐spectrum anti‐parasitic agents are used to improve growth, weight gain, onset of reproductive maturity, and pregnancy rate in cattle and other animals (Larson et al., 1995; Loyacano et al., 2002; Mejia et al., 1999; Powell et al., 2009). However, while parasites frequently inflict fitness costs, there are cases where no apparent cost of parasitism can be detected (Brown et al., 2006; Mayer et al., 2015). Indeed, an apparent lack of costs of parasitism tends to be underreported (Hasik & Siepielski, 2022; Sánchez et al., 2018) and may thus be more common than is appreciated.

In many species, males are more heavily parasitized than females and thus may bear greater costs of parasitism (Aubret et al., 2005; Curtis & Baird, 2008; Dudek et al., 2016; Krasnov et al., 2012; Moore & Wilson, 2002; Zuk & McKean, 1996). When parasites impose a growth cost, sex differences in the intensity of parasitism may contribute to the development of sexual size dimorphism (SSD) (Potti & Merino, 1996). In the present study, we investigated whether ectoparasitic Trombiculid mites reduce growth in a sex‐specific manner and contribute to the development of SSD in eastern fence lizards (*Sceloporus undulatus*).

Eastern fence lizards (*Sceloporus undulatus*) in our study area are heavily parasitized by larval *Eutrombicula alfreddugesi* (Pollock & John‐Alder, 2020), a widespread species commonly known as chigger mites, or simply chiggers. As larvae, chiggers are generalist ectoparasites, which feed on their host's digested, liquefied skin and lymph until they fully engorge, drop off, and molt into the free‐living nymphal stage (Sasa, 1961). In the New Jersey pinelands, it is not uncommon for an individual lizard to host hundreds of chiggers at one time (Pollock & John‐Alder, 2020). Given that lizards mount a local inflammatory response to mites (Goldberg & Bursey, 1991; Goldberg & Holshuh, 1992), chiggers may plausibly impose costs on their lizard hosts, especially when present in great numbers.

During the peak summer activity season of *S. undulatus*, chigger mites are abundant in the environment, all lizards are parasitized by mites (100% prevalence of ectoparasitism), and mite loads on lizards reach their highest levels of the year (Pollock & John‐Alder, 2020). Furthermore, mite loads vary by an order of magnitude among individual lizards, and the rank ordering of the number of mites on individual lizards is significantly concordant from week to week, indicating consistent inter‐individual differences in mite ectoparasitism (Pollock & John‐Alder, 2020). By early July, when juvenile females grow faster than males and sexual size dimorphism (SSD) is developing to its full extent, mite loads are higher on yearling males than on any other age‐sex class (Pollock & John‐Alder, 2020). This is a system in which growth costs of ectoparasitism—and potentially sex‐biased growth costs of ectoparasitism—should be readily apparent because (1) mites are abundant in the environment, (2) all lizards are parasitized by mites at some point during the summer activity season, and (3) mite loads can range from very low to very high (i.e., 0–435). In the present study, we investigated the relationship between mite ectoparasitism and growth rate in yearling juveniles of *S. undulatus*. More specifically, we sought to identify if there is a growth cost to mite ectoparasitism, and if so, if this growth cost contributes to the development of SSD in a female‐larger lizard species due to sex differences in ectoparasitism.

## 2 MATERIALS AND METHODS

We investigated relationships between chigger mite loads and lizard growth rates in yearling juveniles of *Sceloporus undulatus* in the New Jersey Pinelands National Reserve during June and July of 2016 and 2019. We conducted our studies in Colliers Mills Wildlife Management Area (Burlington County, New Jersey, USA; 40.1° N, 74.4° W), which we chose because of its relative abundance of lizards. Our study site was a 0.045 km^2^ patch of oak‐pine forest bounded by open, mowed fields and human‐made sand paths, with a variably open canopy, sparse understory due to prescribed burns, and abundant tree debris on the forest floor.

Eggs in this population are laid from late May into June and begin to hatch in late July. Depending on weather, the neo‐natal activity season for hatchlings can extend into mid‐November. Lizards emerge from winter inactivity as yearlings in March of the next year and grow rapidly to reach the size of reproductive maturity by the end of the first full activity season. Following a second winter of inactivity, lizards re‐emerge as reproductively mature adults at approximately 20 months of age, in the spring of their second full activity season (Haenel & John‐Alder, 2002). Males and females are initially indistinguishable in body size, but sexual divergence in body size occurs most rapidly in yearlings during June/July of the first full activity season, coinciding with rising levels of plasma testosterone in males and faster growth rates in females (Cox et al., 2005; Skelly & John‐Alder, 2002). With the attainment of the size of reproductive maturity at the end of the first full (yearling) activity season, females are approximately 10% larger than males (Haenel & John‐Alder, 2002).

In both years of our study, we marked and recaptured the same lizards repeatedly for measurements of body size and mite loads. In 2016, we made measurements and counted mites on 39 females and 55 males. In 2019, we made measurements and counted mites on 28 females and 20 males. All measurements each year were collected by the same individual (NBP in 2016, HC in 2019) to prevent possible measurement biases in SVL, body mass, or mite counts. Although measurements were performed by a different investigator in 2016 and 2019, it is unlikely that an investigator bias would have influenced calculated growth rates because any differences between measurements would be consistent across the entire growth interval and as such, the calculated growth rates (change in length per time) would not be affected. We captured lizards with a slip‐noose or by hand. Upon capture, we measured snout‐vent length (SVL, mm) using a ruler and body mass to 0.1 g using a Pesola® spring scale. We determined sex by the presence (male) or absence (female) of enlarged post‐cloacal scales, and we unambiguously distinguished yearlings from older age‐classes by their smaller size during the month of June (Haenel & John‐Alder, 2002). Each lizard was given a unique toe clip for permanent identification and a unique dorsal paint mark just anterior to the base of the tail (a region not preferred by mites) for visual identification. To obtain mite loads, we used a 10× hand lens in the field to count the number of mites on each lizard at the time of capture. In 2016, we counted mites on recaptured lizards at weekly intervals from June 9 through July 14, although not all lizards were recaptured every week. In 2019, we counted mites on recaptured lizards every 10–14 days from June 6 through July 18.

In 2019, we assessed the precision of mite counts by comparing the number of mites counted by eye in the field to the number of mites counted by using ImageJ (National Institutes of Health, USA) to analyze photographs of the same lizards taken at the time of mite counts in the field with a Nikon D7200 digital camera and a Sigma 105 mm F2.8 EX DG OS HSM Macro lens. Mite loads obtained by these two methods ranged from 7 to 424 mites/lizard and were highly correlated (Figure S1; *n* = 19; slope: 1.06 ± 0.05, 95% CI: 0.94–1.17, *R* ^2^: .997).

For a direct comparison of 2016 versus 2019, we selected comparable 3‐week periods: 22–24 June through 13–14 July 2016 versus 24–27 June through 15–17 July 2019. These three‐week periods included measurements of SVL and mite counts taken in weeks 3, 4, 5, and 6 of the 2016 study (Pollock & John‐Alder, 2020) and in two intensive recapture efforts of 2019. Intervals between measurements of SVL, thus time intervals for calculating growth rate, invariably differed due to uneven recapture success. In 2016, we demonstrated that growth rate calculated as ((SVL_2_–SVL_1_)/(time in days)) was equal to growth rate computed as the slope of SVL plotted as a function of date of recapture for each lizard (Figure S2; slope: 1.04 ± 0.07, 95% CI: 0.91–1.17, *F*_1,54_ = 253.28, *p* < .001). In 2019, we demonstrated that growth rate calculated as ((SVL_2_–SVL_1_)/(time in days)) was independent of the number of days between measurements of SVL (Figure S3; slope: −0.001 ± 0.003, *F*_1,41_ = 0.12, *p* = .736). To analyze relationships between growth rate and mite load separately for the months of June and July, we averaged each lizard's mite counts taken within the focal periods for each month. For example, June's mite count in 2016 was the average of mites counted in weeks 3 and 4 of that study. We calculated body condition separately in June and in July as the residual of log mass regressed on log SVL using measurements taken in the first and last weeks of the intervals defined above.

### 2.1 Data analysis

We used generalized linear models (proc GLM in SAS version 9.4) to analyze effects of mite load on body condition and also to analyze effects of body size, sex, and year on growth rate and mite load, where the earliest measurement of SVL was used for body size. We log‐transformed growth rate and body size to linearize the relationship between the two. Analyses evaluating the effects of sex and year on mite loads in June and July had logSVL entered as a covariate to remove potentially confounding effects of SVL. Analyses evaluating the effects of mite load on body condition in June and July had sex and year entered as covariates to remove potentially confounding effects. All *p* values were considered significant at the α = 0.05 level.

To determine if mite load impacted growth rate, we used R statistical software (version 4.3.1) to generate a series of generalized linear mixed models (GLMMs; Brooks et al., 2023) evaluating the effects of June SVL, July SVL, June mite load, July mite load, sex, and year on growth rates (in mm/day). Individual lizard ID was included as a random effect, except when its inclusion prevented model convergence. We tested potential interactions between predictor variables using simple linear regressions in an exploratory data analysis to determine if we needed to include interaction terms in our models. Overall, we tested 14 models, including a null model with only individual lizard ID. We analyzed effects of mite loads on overall growth rates separately for June and July because of the substantial difference in environmental mite exposure between the 2 months and because mite loads become higher on yearling males than on females in the first 2 weeks of July (Pollock & John‐Alder, 2020).

## 3 RESULTS

**FIGURE 1:**
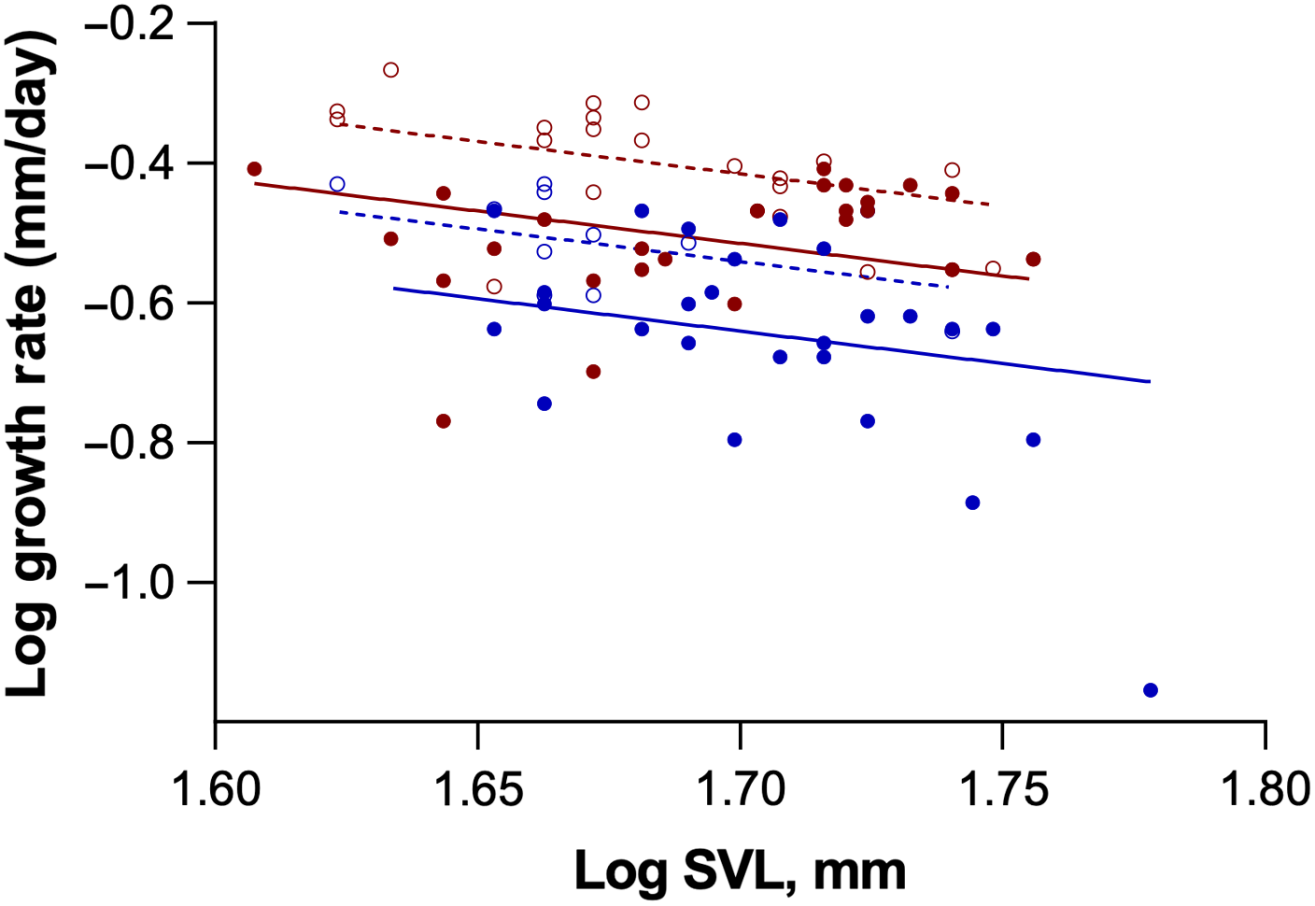
Log growth rate as a function of log June SVL for male and female yearling *S. undulatus* from mark‐recapture studies in 2016 (males: 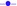; females: 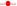) and 2019 (males: 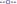; females: 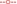). Growth rates were higher in 2019 than 2016, females grew faster than males in both years, and growth rate decreased with increased SVL. The lines are drawn from ANCOVA analyses and are simply for illustrative purposes.

**FIGURE 2:**
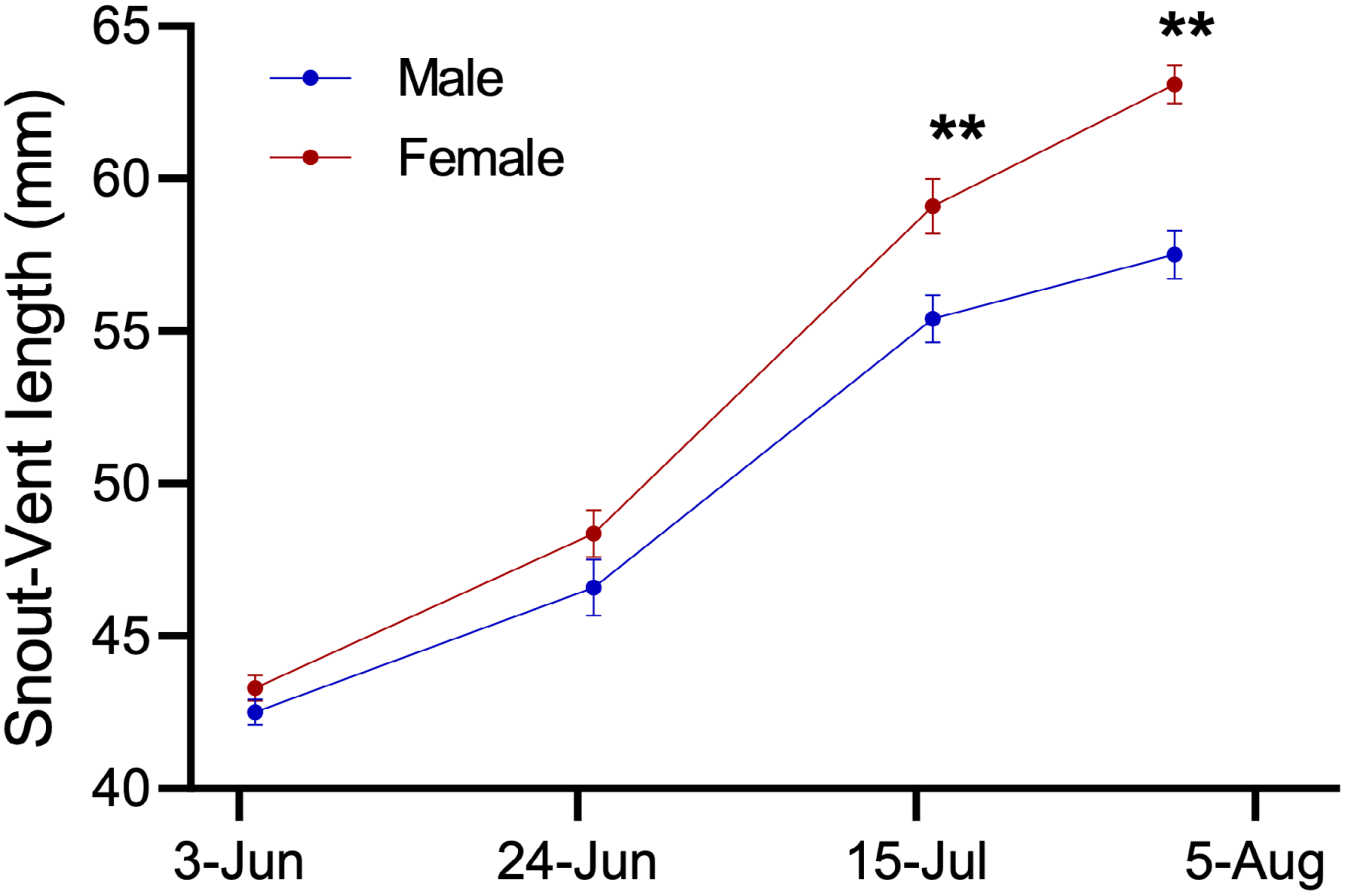
Snout‐vent length of male (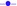) and female (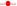) yearling *S. undulatus* across the weeks during the 2019 mark‐recapture study. Growth rate differed between males and females, resulting in the development of significant sexual size dimorphism by 15‐July. Asterisks indicate a significant difference (*p* < .05).

Mite loads, mite load ranges, and growth rates of male and female yearling lizards are summarized in Table 1. Overall, from generalized linear models, growth rate was negatively correlated with SVL, higher in females than in males, and higher in 2019 than in 2016 (Figure 1; overall: *F*_3,82_ = 25.97, *p* < .001; effect of SVL: *F*_1,82_ = 8,65, *p* = .004; effect of sex: *F*_1,82_ = 30.91, *p* < .001; effect of year: *F*_1,82_ = 16.52, *p* = .001). Sex differences in growth rate were similar in 2016 and 2019, resulting in the development of significant sexual size dimorphism (SSD) early in July of both years. In 2019, for example, the difference between female and male SVL, and thus the development of SSD, had become statistically significant by 14 July (Figure 2; *F*_1,25_ = 5.24, *p* = .031).

**TABLE 1:** June and July mite loads, mite load ranges, and growth rates of male and female yearling *S. undulatus* in 2016 and 2019.

Year	Male			Female		
	Mite load		Growth rate (mm/day)	Mite load		Growth rate (mm/day)
	June	July		June	July	
2016	58 ± 4 (55) (range: 1–153)	138 ± 9 (36) (range: 45–302)	0.24 ± 0.01 (32)	60 ± 5 (41) (range: 3–132)	112 ± 9 (36) (range: 10–239)	0.31 ± 0.01 (39)
2019	93 ± 11 (20) (range: 14–180)	119 ± 17 (8) (range: 41–204)	0.31 ± 0.02 (10)	92 ± 13 (28) (range: 2–286)	123 ± 18 (22) (range: 21–315)	0.41 ± 0.02 (20)
*p*	**<.001**	.692	**<.001**	**<.001**	.204	**<.001**

*Note*: Mite loads are mean ± 1 SE (*n*). Significant within‐sex differences between years are indicated in bold.

**FIGURE 3:**
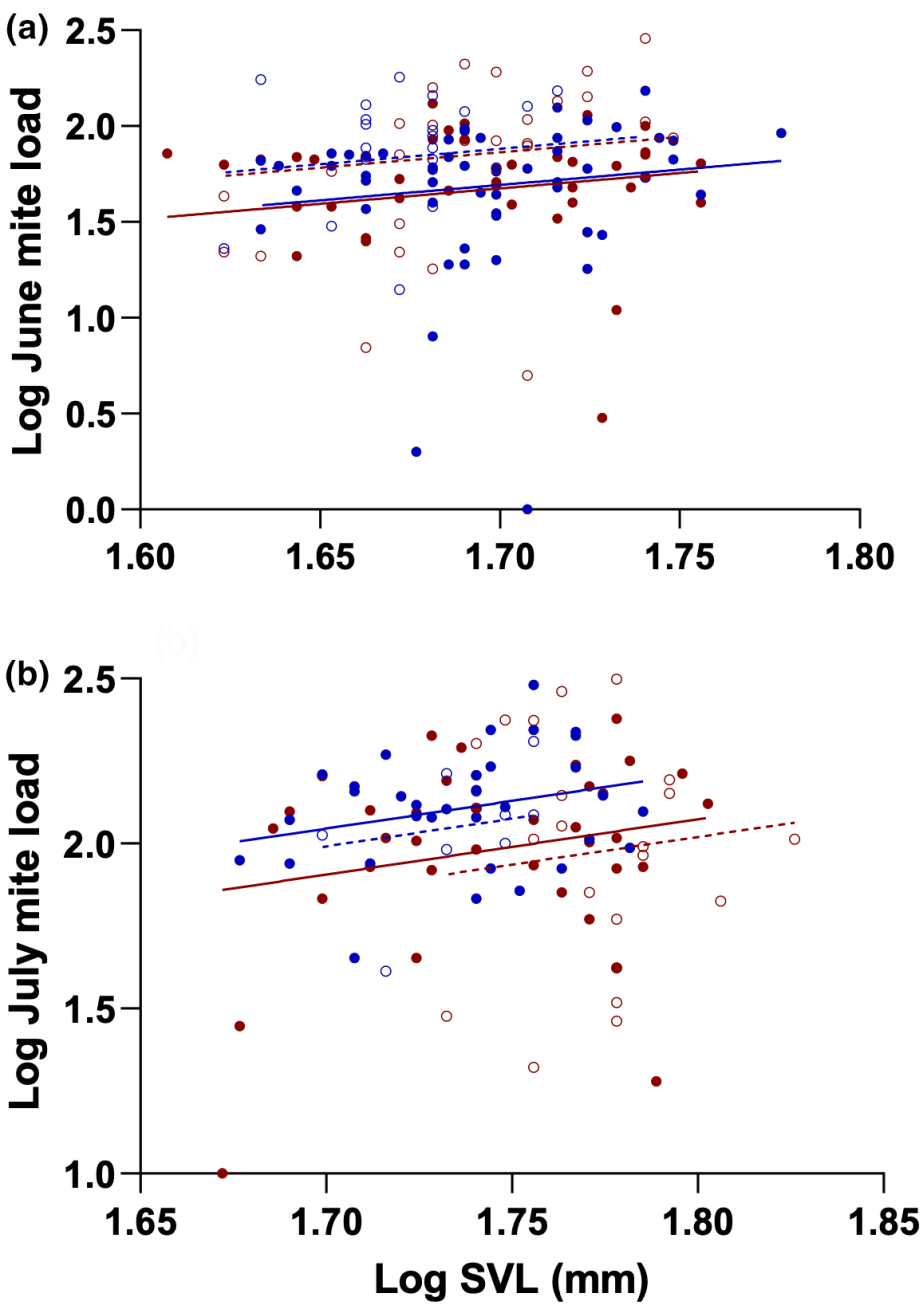
Log mite load as a function of log SVL for male and female yearling *S. undulatus* in June (a) and July (b) from mark‐recapture studies in 2016 (males: 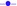; females: 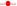) and 2019 (males: 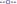; females: 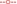). Mite loads were higher in 2019, but only in June. Males had higher mite loads than females, but only in July.

Generalized linear models evaluating the effects of sex and year on mite loads in June and July were overall significant (June: *F*_3,138_ = 3.33, *p* = .022; July: *F*_3,97_ = 3.13, *p* = .029). Mite loads tended to increase with body size (SVL) in both months, but these size effects failed to achieve conventional statistical significance (Figure 3; June: *F*_1,138_ = 3.17, *p* = .077; July: *F*_1,97_ = 3.78, *p* = .055). Controlling for SVL, mite loads were significantly higher on males than on females in July (Figure 3b; *F*_1,97_ = 6.63, *p* = .011), but not in June (Figure 3a; *F*_1,138_ = 0.09, *p* = .764), and were higher in 2019 than in 2016 in June (Figure 3a; *F*_1,138_ = 8.32, *p* = .004) but not in July (Figure 3b; *F*_1,97_ = 0.83, *p* = .364).

**FIGURE 4:**
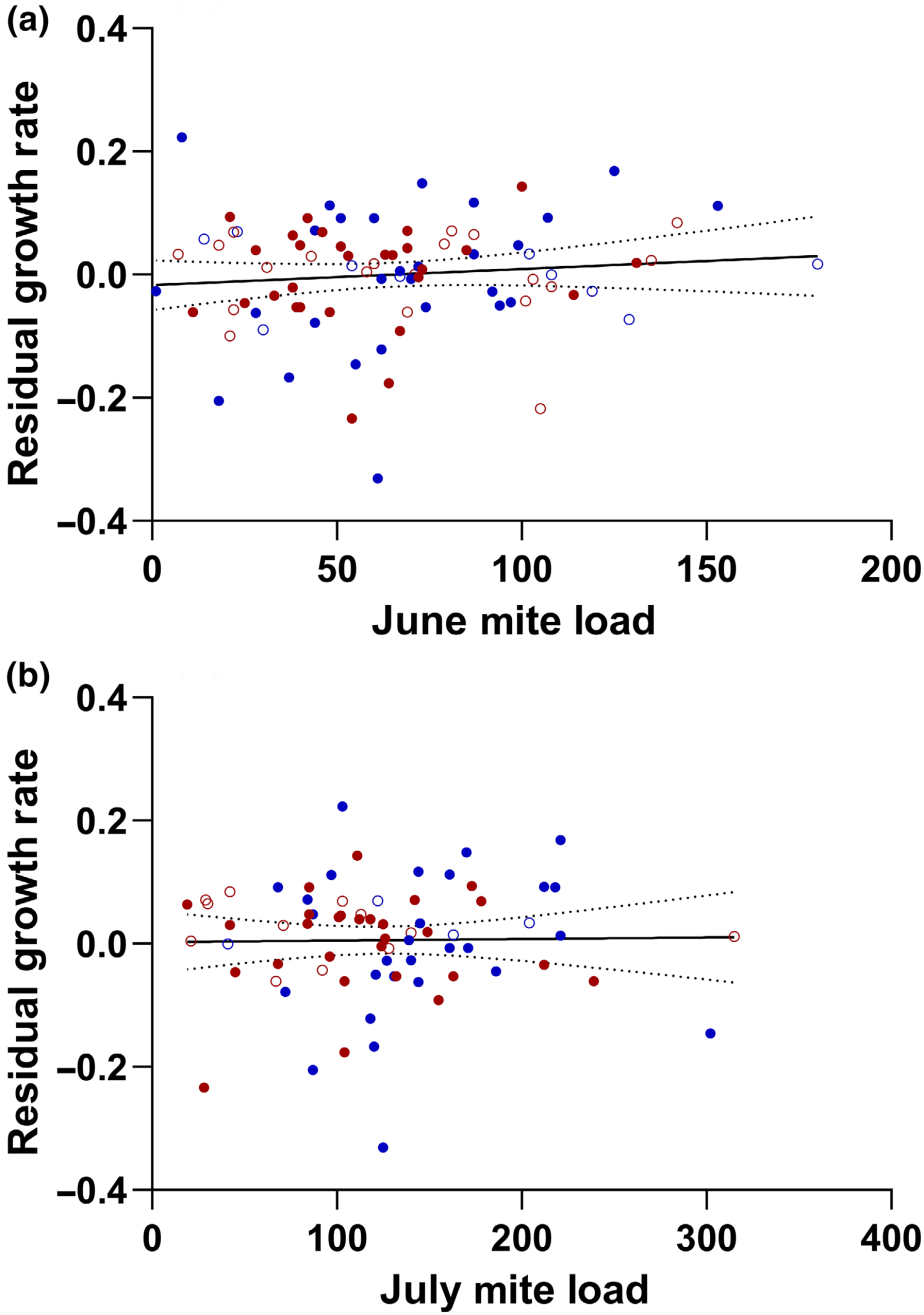
Residual growth rate (mm/day) as a function of mite load for male and female yearling *S. undulatus* from combined 2016 (males: 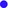; females: 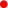) and 2019 (males: 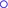; females: 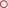) studies for June (a) and July (b). Growth rates of males and females were not significantly affected by mite load in either month of either year. The solid lines are drawn from linear regression models and are simply for illustrative purposes. Dashed lines are 95% confidence bands.

**FIGURE 5:**
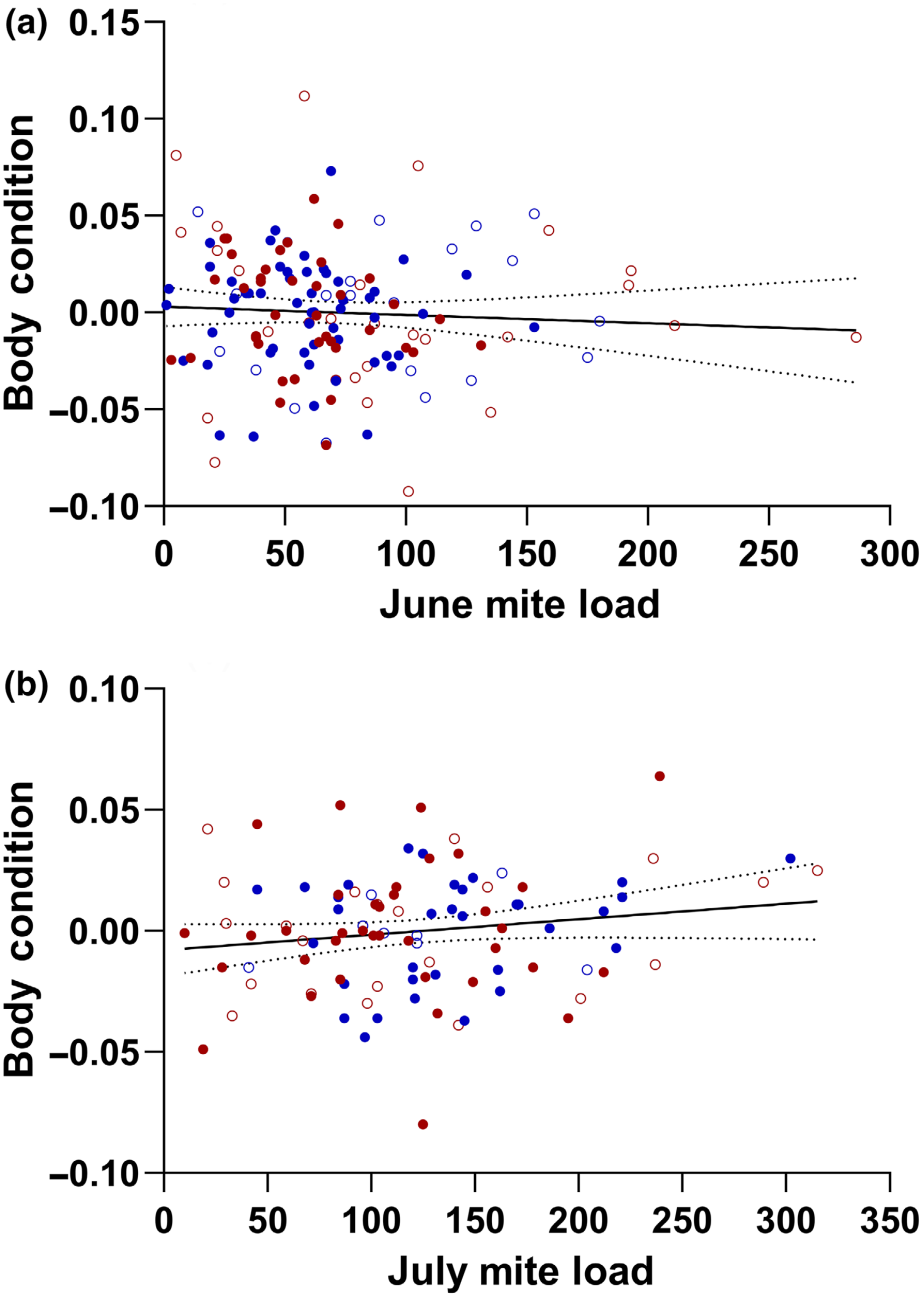
Body condition as a function of mite load for male (blue) and female (red) yearling *S. undulatus* from combined 2016 (males: 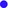; females: 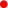) and 2019 (males: 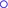; females: 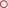) studies for June (a) and July (b). In June, lizards with higher mite loads tended to have slightly lower body condition. In July, however, body condition was not significantly affected by mite load. The solid lines are drawn from linear regression models and are simply for illustrative purposes. Dashed lines are 95% confidence bands.

Overall, mite load does not appear to impact growth rate (Figure 4). None of the GLMM models that included mites as a variable had mites as a significant predictor of growth rate, especially when sex and year were included in the model. Model outputs are summarized in Table 2. We also tested models with interaction terms for sex and year, sex and SVL, and SVL and mites, but none of those models showed the interaction terms as significant predictors of growth rate or improved the overall fit of a model. Similarly, mite load had no significant effects on body condition in June or July (Figure 5; June: *F*_3,138_ = 0.18, *p* = .909; July: *F*_3,96_ = 0.89, *p* = .452).

**TABLE 2:** Values of Akaike's information criterion (AIC) for the 14 potential models describing growth rate.

Model	AIC	ΔAIC	Residual deviance
June SVL* + Sex* + Year*	−194.9	0	−204.9
June SVL* + June Mites + Sex* + Year* + ID*	−191.3	3.6	−205.3
Sex* + Year* + ID*	−188.5	6.4	−198.5
June Mites + Sex* + Year* + ID*	−188.2	6.7	−200.2
July Mites + Sex* + Year* + ID*	−187.6	7.3	−199.6
July SVL + Sex* + Year* + ID*	−186.5	8.4	−198.5
July SVL + July Mites + Sex* + Year* + ID*	−185.6	9.3	−199.6
Year* + ID*	−167.7	27.2	−175.7
Sex*	−163.1	31.8	−169.1
June SVL* + ID*	−145.8	49.1	−153.8
July Mites	−145.7	49.2	−152.7
ID*	−139.3	55.8	−145.3
June Mites + ID*	−138.9	56.0	−146.9
July SVL	−142.7	60.6	−148.7

*Note*: An * indicates significant predictors of growth rate within the models. Any time mites were included in a model, they were not shown to be a significant predictor of growth rate. Delta AIC (ΔAIC) is the difference between the specific model and the best‐performing model.

## 4 DISCUSSION

In the host–parasite system we studied, chigger mites (*E. alfreddugesi*) are highly abundant in the environment, and all age‐ and sex‐classes (adults and juveniles; males and females) of *S. undulatus* are heavily parasitized (Pollock & John‐Alder, 2020). At any given mite load, females grow more quickly than males, which could suggest that mites impose a sex‐biased growth cost. However, we found no evidence that mites impose a growth cost, despite very high mite loads and consistent week‐to‐week rank ordering of mites on individual lizards (Pollock & John‐Alder, 2020). Furthermore, across cohorts, both growth rate and mite loads were higher in 2019 than in 2016, which is opposite the prediction if mites imposed a growth cost. Even in captivity in the absence of mites, SSD develops because females grow faster to become larger than males (Duncan et al., 2020). It follows that the temporal correlation between the development of SSD and the seasonal attainment of sex‐biased mite loads in July does not reflect a sex‐biased growth cost of chiggers.

The general narrative on whether mite parasitism affects growth has been mixed. Studies on *Norops polylepis* (Schlaepfer, 2006) and *Urosaurus ornatus* (Paterson & Blouin‐Demers, 2020) have reported no effects of mite load on growth rate and survivorship, while a study on *Gallotia atlantica* (García‐Ramírez et al., 2005) found no effect of mite load on body condition. In *S. virgatus*, early studies found no effects of mite load on growth rate, body condition, or survivorship (Abell, 2000; Smith, 1996). However, a later study by Cox and John‐Alder (2007) found a strong negative correlation between mite load and growth rate. This study involved experimental manipulation of testosterone and statistical procedures to factor out effects of treatment group, but the results suggest that there are growth costs of mite ectoparasitism. Similarly, Curtis and Baird (2008) found evidence of a growth cost associated with heavy parasitism in collared lizards (*Crotaphytus collaris*) during the peak growing season. This study reported high mite loads, an average of 178.3 ± 65.2 mites on yearling male lizards, which are comparable to what we found on *S. undulatus* in the present study. It is worth mentioning that the mite loads on lizards in the studies finding no growth cost of mite parasitism were substantially lower than those on *S. undulatus* in the present study (*S. undulatus*: 0–435 mites; *U. ornatus*: 0–120 mites; *N. polylepis*: 0–37 mites, *G. atlantica*: 1–28 mites). Despite our higher mite loads, however, we still found no effect on growth.

Overall, the inconsistent narrative regarding potential growth and fitness costs of mites suggests that effects of, or correlates of, mite parasitism can depend on which life history trait is being investigated and the timing of the studies, as ecological conditions (i.e., temperature, precipitation, food abundance, etc.) can fluctuate across the years and have different effects on life history traits. Tolerance to parasites is greater in populations that experience higher variation in environmental conditions (Ferris & Best, 2019). A study on mockingbirds (*Mimus parvulus*) showed that tolerance to parasites and effects of parasites varied through the years based on rainfall and food abundance. The effects of parasites on the hosts were not because the parasites caused more damage, but were because the hosts could not compensate for the parasite‐induced costs (McNew et al., 2019). In our study, sampling error may have also played a role as our sample populations may not be representative of the overall population. Furthermore, effects of mite parasitism can differ not only among host species but also between populations of the same species as observed with the studies on *S. virgatus* (Abell, 2000; Cox & John‐Alder, 2007; Smith, 1996). As such, it is difficult to envision an overall evolved strategy to mite parasitism at a species level.

While not a major consideration in our study, we posit that future studies on the costs of mite parasitism in lizards should consider whether the host population in the system would be expected to have evolved a strategy of resistance or tolerance towards the parasites in their study design. The ecological relationship between the populations of *S. undulatus* and *E. alfreddugesi* in this study (i.e., high environmental mite abundance and high host mite loads) is typical of a host–parasite system in which the host would be expected to have evolved tolerance to parasites (Pollock & John‐Alder, 2020; Råberg et al., 2009). Hosts can evolve tolerance so effectively that the reduction in fitness caused by parasites may not be measurable (Råberg, 2014) and studies that do not find a growth cost associated with parasitism may be examining systems where hosts have evolved a strategy of tolerance towards parasites, as appears to be the case for lizards and mites. For example, it has been hypothesized that mite pockets evolved as a compensatory mechanism for reducing the harm caused by mite ectoparasitism because they concentrate mites in areas better equipped to heal and where they do not interfere with other functions, such as movement and vision (Arnold, 1986; de Carvalho et al., 2006; Reed, 2014; Salvador et al., 1999). Tolerance can be quantified using the slope of the relationship between fitness (or its proxy) and parasite burden (Burgan et al., 2019). Given that we found a slope of zero in the relationship between growth rate and mite load, our population of eastern fence lizards is highly tolerant of chiggers by the criteria we evaluated (Råberg, 2014; Råberg et al., 2007). Ornate tree lizards (*Urosaurus ornatus*) have also been found to be highly tolerant of mite parasites (Paterson & Blouin‐Demers, 2020). Overall, host–parasite systems in which hosts appear to be highly tolerant of the parasite are not well‐suited for investigating sex‐biased costs of parasitism because any costs of parasitism will not be easily measurable.

If chigger mites in our study population are in fact extracting a substantial cost from yearling *S. undulatus*, the cost may be at the expense of traits and functions other than growth. Rapid growth is expected to be under strong selection to ensure that yearlings grow to the minimum size of reproduction by the end of their first full activity season (Adolph & Porter, 1996; Haenel & John‐Alder, 2002). Furthermore, female body size is positively correlated with clutch size, suggesting strong fecundity selection on body size and thus yearling growth (Angilletta et al., 2001; Brandt & Navas, 2011; Cox et al., 2003; Haenel & John‐Alder, 2002; Jiménez‐Arcos et al., 2017). Thus, potential explanations for the absence of growth costs could include that costs of parasitism are (1) traded off against other functions, (2) too small to be of any consequence, or (3) compensated by increased dietary consumption. We now consider each of these possibilities.

If mites do impose costs on juveniles of *S. undulatus*, it seems likely that a potential growth cost might be traded off against other traits (Adolph & Porter, 1996). For example, reduced activity is one of the apparent costs of parasitism in common lizards (*Lacerta vivipara*) and Western fence lizards (*Sceloporus occidentalis*) (Clobert et al., 2000; Megia‐Palma et al., 2020). In the present study, we did not quantify a specific measure of activity in *S. undulatus*. However, in a 2019 companion study conducted at our study site and on the same population of *S. undulatus* we found that home range area is greater in yearling males than in females (1101 ± 327 m^2^ vs. 233 ± 62 m^2^) and is not correlated with growth rate (*F*_2,1_ = 0.14, *p* = .708), even while mite loads are greater in males than in females (Conrad, 2019; Yawdoszyn, 2019). Home range area can be used as a proxy for energy expenditure on activity to assess the possibility of a trade‐off against activity instead of growth (Christian & Waldschmidt, 1984; Hews, 1993; John‐Alder et al., 2009; Main & Bull, 2000). Thus, our evidence suggests that mites do not impose an activity cost of mite ectoparasitism. However, while home range area can be used as a first approximation of activity, a study of the close congener *S. occidentalis* reported that lizards infected with malarial parasites maintained the same home range area as unparasitized lizards but showed reduced daily activity (Schall & Houle, 1992). Thus, time‐intensive focal observations would be required for a definitive determination of whether mites impose an energetic cost on *S. undulatus* as seen through reduced activity.

Lacking evidence of costs of mite parasitism, it is possible that mites, even in high numbers, may not impose a measureable energetic cost, either directly or indirectly. We estimated the energetic cost of a single chigger mite to be approximately 0.004 J/day using estimates of chigger mite body size (body length = 0.4 mm, body mass = 0.9 μg; Johnson & Strong, 2000) and the metabolic scaling exponent of 0.75 (West et al., 2002). Taking the mean mite loads of yearling male lizards in June 2016 (58 mites), July 2016 (138 mites), June 2019 (93 mites), and July 2019 (119 mites), the average energetic costs of mite ectoparasitism for yearling male lizards would have been 0.232 J/day in June 2016, 0.552 J/day in July 2016, 0.372 J/day in June 2019, and 0.476 J/day in July 2019. For comparison, the energetic cost of growth is about 130 J/day for yearling males (Cox et al., 2005). Thus, in the aggregate, mites imposed an energetic cost that was a mere 0.002%–0.004% of the energy cost of growth.

The energetic cost of direct energy extraction by mites is unlikely to be detectable as a growth cost because of its low value. These estimations, however, do not account for the potential indirect energetic costs of mounting an inflammatory immune response or the accompanying stress to the lizard in response to mite ectoparasitism, which we did not measure. However, potential energetic costs of immune or stress responses are complex. A meta‐analysis by van der Most et al. (2011) found that selection for growth compromises immune function, but selection for immune function does not appear to affect growth. This suggests that the costs of growth are large relative to the costs of immune function. A study of parasites in side‐blotched lizards, *Uta stansburiana*, found increased immunocompetence associated with parasitism, but no other measurable costs (Spence et al., 2017). Despite the lack of measurable costs in *U. stansburiana*, it can be costly for a host to up‐regulate their immune system in response to parasites, forcing life‐history trade‐offs over time (French et al., 2009; Lochmiller & Deerenberg, 2000; Sheldon & Verhulst, 1996). Overall, while there may be some energetic cost to immunity, those costs are likely too small to be detectable as costs to growth. However, more research is certainly needed in this area.

Finally, growth and body condition may not have been associated with mite ectoparasitism in *S. undulatus* because environmental conditions favorable for ectoparasitism were at the same time favorable for abundant prey, including other arthropods and possibly even mites themselves. Thus, even while they were heavily parasitized by mites, lizards would be enabled to increase their energy consumption and compensate for costs of a high parasite load. The spring of 2019 had more precipitation and higher temperatures than the spring of 2016 (http://climate.rutgers.edu/stateclim_v1/nclimdiv/), and we saw an increase in both environmental mite abundance and lizard growth in 2019 as compared to 2016. Increased precipitation would have increased soil moisture and humidity, which are positively correlated with the abundance of arthropods, including mites (Kardol et al., 2011; Prather et al., 2020; Zippel et al., 1996). Temperature has also been shown to be positively correlated with arthropod abundance (Lessard et al., 2011). Mite activity is increased by a combination of moderately high temperatures and high humidity (Clopton & Gold, 1993). At the same time, higher temperatures can create opportunities for increased daily activity in *S. undulatus*, while also contributing to increased growth rates and faster maturation (Adolph & Porter, 1996). Greater arthropod abundance may have increased food availability for *S. undulatus* as well, facilitating increased growth. A greater understanding of the ecological interactions between *S. undulatus*, mites, and other arthropods could show if lizards increase their feeding rates on other arthropods due to increased mite ectoparasitism.

In summary, there are many possible explanations for why we did not find a growth cost associated with mite parasitism in either sex of *S. undulatus* in this study. Even when males had lower mite loads than females, females still grew to larger sizes. So, it is safe to conclude that sex‐biased costs of mite parasitism are not contributing to the development of SSD in our study population of *S. undulatus*. The lizards in our study population appear to be highly tolerant of mite ectoparasitism, but definitive experimental studies are required to rule out various trade‐offs and effects of environmental food abundance. As such, while our study population also exhibits the common male‐biased pattern of ectoparasitism, it also shows that we should be careful in making conclusions about the implications of sex differences in parasite intensity and prevalence.

## AUTHOR CONTRIBUTIONS

**Nicholas B. Pollock:** Conceptualization (equal); data curation (equal); formal analysis (supporting); funding acquisition (supporting); investigation (equal); methodology (equal); project administration (equal); supervision (equal); writing – original draft (equal); writing – review and editing (equal). **Hailey Conrad:** Conceptualization (supporting); data curation (equal); formal analysis (supporting); investigation (equal); methodology (equal); project administration (equal); writing – original draft (equal); writing – review and editing (equal). **Henry John‐Alder:** Conceptualization (equal); data curation (equal); formal analysis (lead); funding acquisition (lead); investigation (equal); methodology (equal); project administration (equal); supervision (equal); writing – original draft (supporting); writing – review and editing (supporting).

## Supporting information


Appendix S1.

Appendix S2.

Appendix S3.

Appendix S4.

Appendix S5.

## Data Availability

The data used in this study have been archived in the Dryad Digital Repository (https://doi.org/10.5061/dryad.tb2rbp076).
