# Supplementary material for: Chigger mite (Eutrombicula alfreddugesi) ectoparasitism does not contribute to sex differences in growth rate in eastern fence lizards (Sceloporus undulatus)
Source: Ecol Evol. 2023 Oct 11;13(10):e10590. doi: 10.1002/ece3.10590 (PMC10565727; doi:10.1002/ece3.10590)
Supplement: Supplementary file 4 — Appendix S4. [file ECE3-13-e10590-s003.docx]

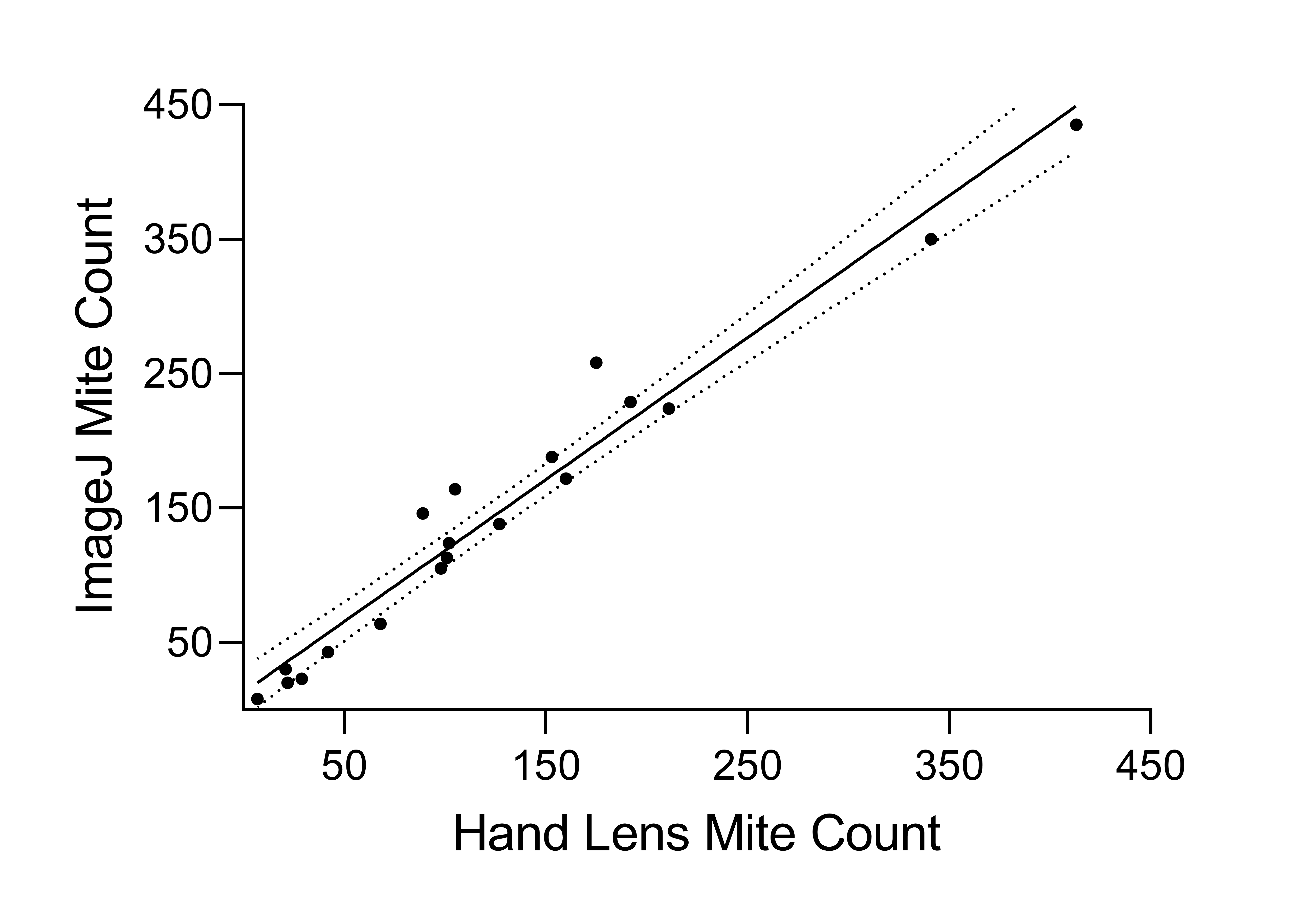


**Figure S1.** Accuracy of mite counts using a hand lens and counting by eye compared to using a digital camera with macro lens and ImageJ software. Mite loads determined by these two methods ranged from 7 to 424 mites/lizard and were highly correlated (n = 19; slope: 1.06 ± 0.05, 95% CI: 0.94–1.17, R^2^: 0.997).


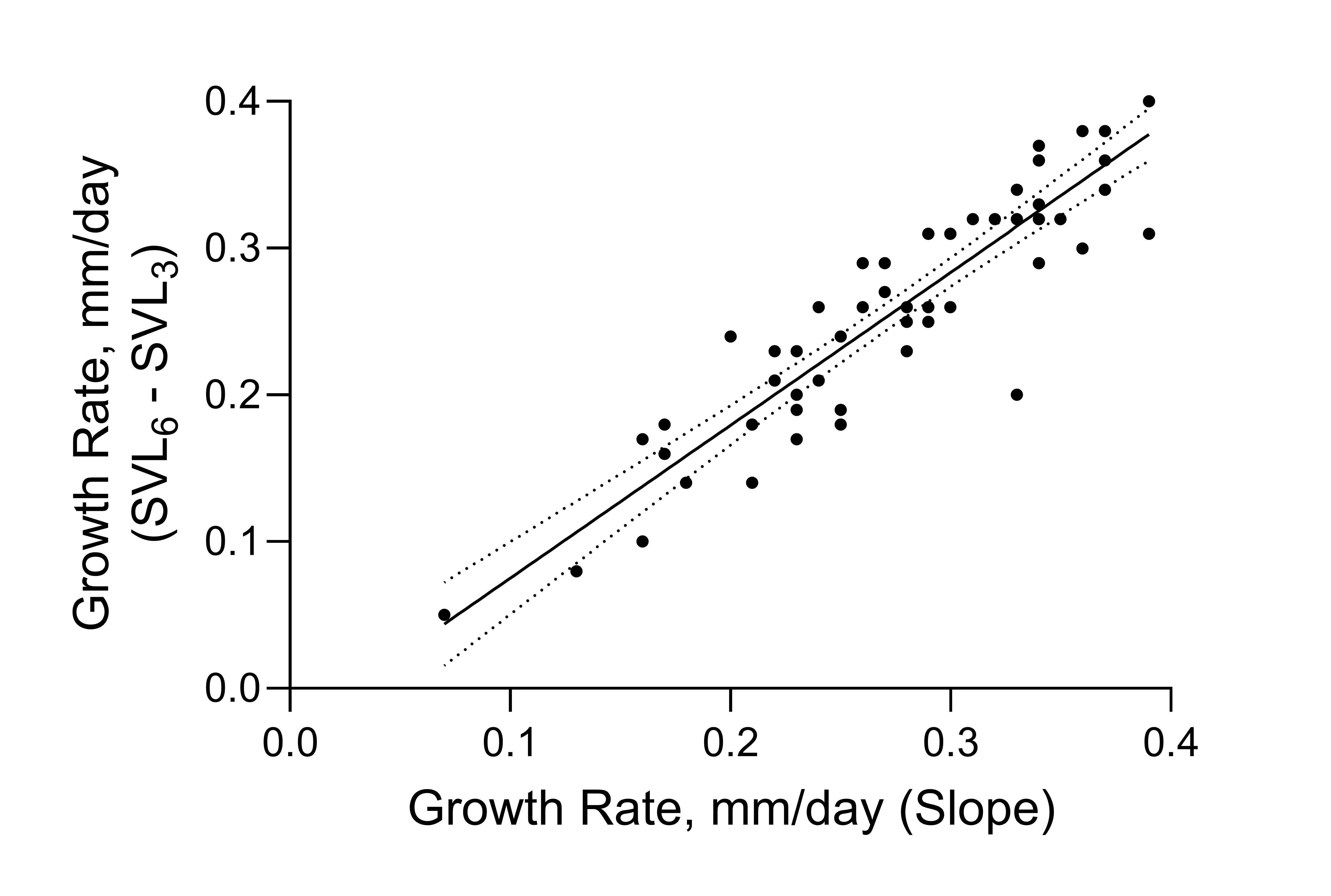


**Figure S2.** In 2016, we calculated growth rate as ((SVL_2_ -SVL_1_) / (time in days)), which was equal to growth rate computed as the slope of SVL plotted as a function of date of recapture for each lizard (slope: 1.04 ± 0.07, 95% CI: 0.91–1.17, F_1,54_ = 253.28, *p* < 0.001).


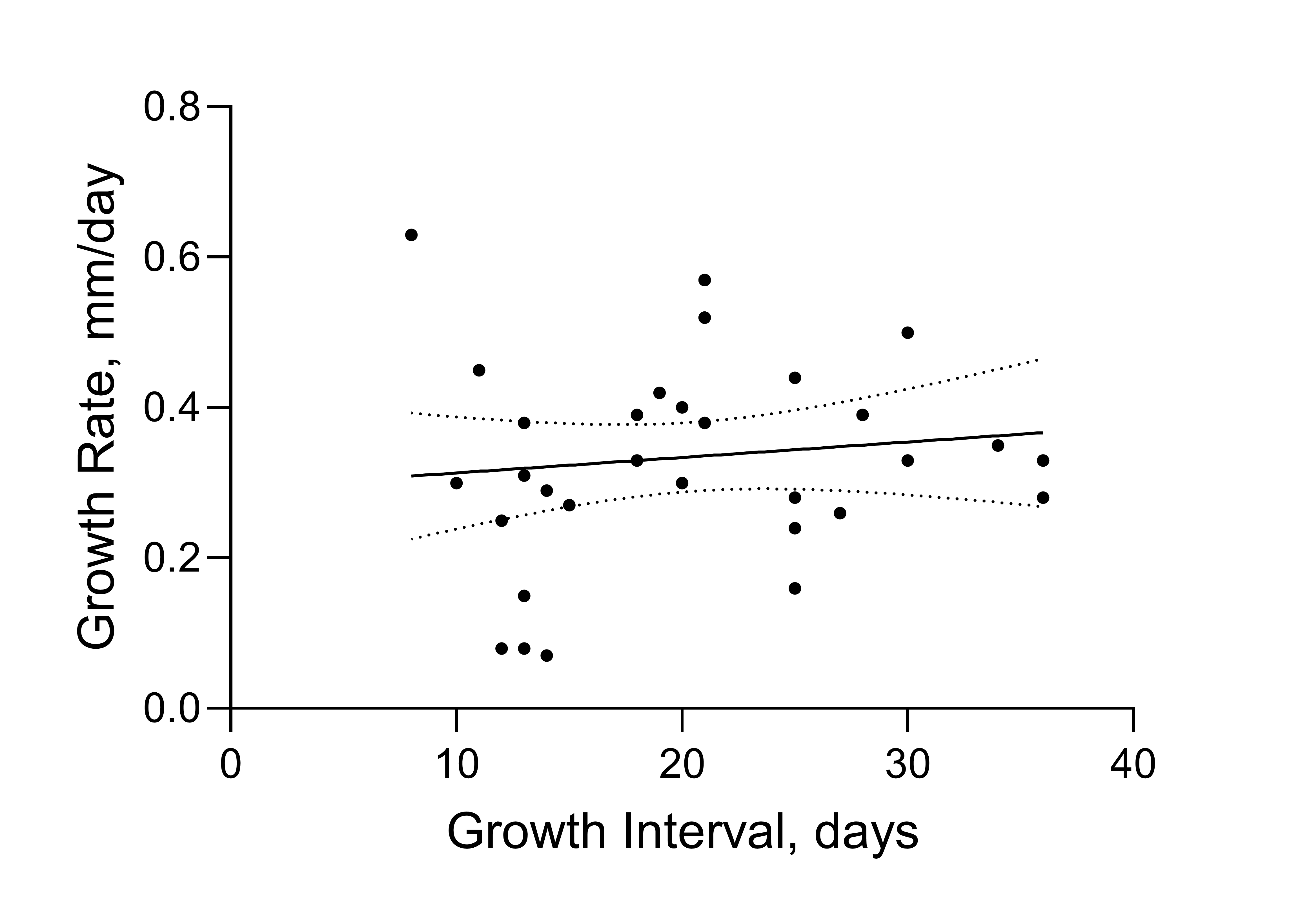


**Figure S3.** In 2019, we calculated growth rate as ((SVL_2_ -SVL_1_) / (time in days)), which was independent of the number of days between measurements of SVL (slope: -0.001 ± 0.003, F_1,41_ = 0.12, *p* = 0.736).
